# Socio-Emotional Experiences and Wellbeing of Deaf and Hard of Hearing Children and Their Parents before and during the COVID-19 Pandemic

**DOI:** 10.3390/children10071147

**Published:** 2023-06-30

**Authors:** Alanna N. Gillespie, Libby Smith, Daisy A. Shepherd, Jessica Xu, Rija Khanal, Valerie Sung

**Affiliations:** 1 Prevention Innovation, Murdoch Children’s Research Institute, Parkville, VIC 3052, Australia; alanna.gillespie@mcri.edu.au (A.N.G.); libby.smith@mcri.edu.au (L.S.); jessica.xu@mcri.edu.au (J.X.);; 2 Centre for Community Child Health, The Royal Children’s Hospital, Parkville, VIC 3052, Australia; 3 Department of Paediatrics, The University of Melbourne, Parkville, VIC 3010, Australia; daisy.shepherd@mcri.edu.au; 4 Clinical Epidemiology and Biostatistics, Murdoch Children’s Research Institute, Parkville, VIC 3052, Australia

**Keywords:** deaf, hard of hearing, socio-emotional, COVID-19, pandemic, adverse childhood experience

## Abstract

Deaf and hard of hearing (DHH) children in Victoria, Australia, were exposed to strict public health restrictions, including sustained lockdowns, during the COVID-19 pandemic. DHH children have higher health and socio-emotional needs than their hearing peers. We aimed to (1) describe the socio-emotional experiences of DHH children and their parents and (2) compare child and parent socio-emotional wellbeing, before and during the COVID-19 pandemic. Between May and September 2020, 497 (62%) parents of DHH children from the Victorian Childhood Hearing Longitudinal Databank completed an online survey. Measures were drawn from the CoRonavIruS Health Impact Survey (CRISIS) v3.0. Data were summarized using descriptive statistics to compare outcomes before and during the pandemic. Parents reported their children to have more negative socio-emotional wellbeing (mean emotions/worries score, EWS, changed from 0.76 pre-pandemic to 1.10 during the pandemic, mean difference 0.34, 95% CI: 0.28 to 0.39), regardless of the type or severity of hearing loss. Parents also had more negative socio-emotional wellbeing (mean EWS changed from 1.05 pre-pandemic to 1.43 during the pandemic, mean difference 0.38, 95% CI: 0.31 to 0.44). Negative socio-emotional experiences co-occurred with large social changes during the pandemic. Additional services should support the socio-emotional wellbeing of DHH children during significant adverse childhood experiences.

## 1. Introduction

Socio-emotional development is an essential component of optimal child development [1]. It allows children to interact with others via reciprocal social interactions [2] and is crucial for the development of relationships with family and peers. However, deaf and hard of hearing (DHH) children [3] often experience socio-emotional difficulties, with evidence suggesting that up to 41% of deaf children aged 4–18 years have emotional and behavioral problems [4], rates that are approximately two times higher than problems reported in hearing children [5,6,7,8,9,10]. These difficulties may be compounded by exposure to adverse childhood experiences (ACE).

Research suggests that the recent COVID-19 pandemic, which spread globally in 2020 [11] and resulted in social restrictions on day-to-day life in Australia, is an ACE for some [12], with resulting impacts on socio-emotional development. In Australia, International borders were shut in March, 2020, leading to low case numbers throughout 2020 and 2021 until vaccination was widely available [13,14]. Despite this, the state of Victoria experienced a prolonged second wave of cases during 2020. In the absence of available vaccinations, the government imposed stay-at-home orders (lockdowns), restrictions on gatherings, school closures, face masks, workplace orders (business closures and working from home), and a range of other public health measures to reduce the spread of COVID-19 [15]. The pandemic resulted in financial hardships, social isolation, and increased psychological distress [16], as well as impacting adolescent mental health, with three-quarters of participants from a study of 760 Australian adolescents aged 12–18 years reporting poorer mental health during the pandemic [17]. A global meta-analysis of 29 studies suggests negative impacts on mental health from the ongoing pandemic were widely experienced with children and adolescents reporting anxiety and depressive symptoms at almost double pre-pandemic rates [18].

COVID-19′s impacts on children’s socio-emotional development are likely even more pronounced in children with chronic health needs and disabilities [19]. However, research on the socio-emotional health and well-being of DHH children during the COVID-19 pandemic is limited. A qualitative study suggested significant challenges for 37 Saudi Arabian DHH students with home schooling/distance education [20]. One explanation is that DHH children and their families may be affected differently to typically hearing children by online education, mask wearing, and social isolation policies that were in place during the pandemic, particularly if they would normally rely on assistive technology or extra aid in the classroom to support their learning [21]. A survey of 416 educators working with DHH children in the United States between September and December 2020 outlined significant auditory signal attenuation and loss of visual cues from face masks as well as reduced access to sign language interpreters and lack of captioning of online learning material causing significant challenges for DHH students [22]. Whilst there is a general understanding that DHH children often experience mental health challenges [23] at higher rates than the general population, to date there is limited evidence quantifying the socio-emotional changes DDH children experienced during (compared to before) the COVID-19 pandemic. Understanding the impacts of the pandemic on DHH children and their families will help ensure they receive targeted support and services.

In a population of DHH children and their parents/caregivers from Victoria, Australia, we aimed to (1) describe the socio-emotional experiences of DHH children and their parents and (2) compare child and parent socio-emotional wellbeing, before and during the COVID-19 pandemic.

## 2. Materials and Methods

### 2.1. Study Design, Recruitment, and Follow-Up: Overview of the VicCHILD Study

The Victorian Childhood Hearing Longitudinal Databank (VicCHILD) is a statewide population-based databank that aims to identify the longitudinal outcomes of DHH children in the state of Victoria (population around 6.8 million), Australia. It commenced in 2012 with ongoing data collection and is open to all Victorian children with permanent hearing loss [24]. Parents (including caregivers) are invited to participate through the Victorian Infant Hearing Screening Program (VIHSP), which offers a hearing screen to 99.5% of Victorian babies in the days after birth, and the Caring for Hearing in Children Clinic (CHIC) at Melbourne’s Royal Children’s Hospital. Children are eligible for VicCHILD if they are identified as having hearing that is not normal and hearing loss is defined as permanent. All children who are recruited into the VicCHILD databank have permanent hearing loss confirmed via audiological assessment and parental report with hearing loss thresholds defined as follows: <20 dB HL normal; 20–40 dB HL mild; 40–60 dB HL moderate; >60–90 dB HL severe; >90 dB HL profound in the better hearing ear. With consent, parents complete surveys at baseline and subsequent surveys and assessments at key child developmental stages. The majority (92%) of parents consent to being contacted about future research projects. Further details about VicCHILD’s methodology are described elsewhere [24].

In late May 2020, all parents of participating VicCHILD children who had previously provided consent to be contacted about other research projects were emailed an invitation to complete an online survey, with close of survey responses by mid-September 2020. The beginning of this survey period corresponded to public health recommendations to maintain physical distancing, practice hygiene and self-isolation when ill, and work from home, if possible. Towards the end of the survey period from July 2020, Victoria was under ‘lockdown’ with only three reasons to leave home (exercise, work if it could not be completed from home, and shopping for essential groceries).

Survey measures were drawn from the CoRonavIruS Health Impact Survey (CRISIS) v3.0 [25], which asked about child and family experiences 3 months before and during the pandemic. The CRISIS was developed during the earliest stages of the COVID-19 pandemic to capture the events and challenges that were occurring for children and their parents [25]. The survey domains included demographics, physical health, COVID-19 exposure, and aspects of socio-emotional experiences including changes to daily behaviors (child learning or care arrangements), parent employment, financial problems, daily activities (sleep, exercise, screen time), family life, and socio-emotional wellbeing. Socio-emotional wellbeing was measured using the emotions/worries scale 8-item mean score which measured the following for the child: worries, happy versus sad, relaxed versus anxious, fidgety or restless, fatigued or tired, able to concentrate or focus, irritable or easily angered, and feelings of loneliness [25]. Responses were on a 5-point Likert scale between 0 and 4, with 0 representing the best outcome, i.e., least sad, and 4 representing the poorest outcome, i.e., most sad [25]. The CRISIS v3.0 was also used to measure the impacts of the COVID-19 pandemic on the socio-emotional wellbeing of children in other large longitudinal population studies at the Murdoch Children’s Research Institute [26].

VicCHILD study-designed questions from previous waves of data collection were repeated in this survey (demographics). The Socio-Economic Indexes for Areas Index of Relative Socio-Economic Disadvantage (SEIFA) was used to measure socioeconomic disadvantage from the address of the main caretaker. It is a composite census-based measure summarizing the social and economic conditions of Australian neighborhoods (national mean 1000, SD 100, where higher values represent less disadvantage) [27]. We also calculated a measure of geographical remoteness from the address of the main caretaker using Australian Standard Geographical Classification (ASGR) Remoteness Area Correspondences [28]. The survey questions/responses are summarized in Supplementary Tables S1 and S2. Within this survey, we also asked VicCHILD parents about their child’s frequency of service use before and during the pandemic and use of telehealth (e.g., parent views on the use of telehealth, quality of telehealth); these results are reported elsewhere [29].

### 2.2. Data Analysis

For aim 1, we summarized survey responses using descriptive statistics. Continuous variables were reported as means and standard deviations and categorical variables as proportions. For aim 2, where continuous data were available for the same measure before and during the pandemic, a paired *t*-test estimated the mean difference between the two time points, with the corresponding 95% confidence intervals (CI) presented. For categorical variables, comparison between the distributions before and during the pandemic were made descriptively, considering the magnitude of any observed differences using a Stuart Maxwell test.

## 3. Results

### 3.1. Demographics

A total of 806 parents were invited to fill out the survey, with 497 (62%) completing it. There were minimal differences in child and family characteristics between responders and non-responders (Table 1). Responders lived in areas representing slightly higher socio-economic status as measured by the Socio-Economic Indexes for Areas (SEIFA, responder mean: 1013.5, SD: 65.1 vs. non-responder mean: 1006, SD: 68.5). Almost one-fifth (19%) of respondents lived in a remote area. Most responders were mothers (94%), spoke English as the first language (79%), completed high school (89%), and held a tertiary qualification (59%). Almost all (90%) had a partner living with them at the time of completion and families had an average of four people living in the home.

The mean age of children was 6.5 years (range 0 to 17 years; SD 4.0 years) at the time of data collection. Most had sensorineural (68%), bilateral (65%) hearing loss, with a range of severity from mild to profound. Over a third (39%) had at least one other comorbidity/health need in addition to hearing loss.

### 3.2. COVID-19 Exposure

None of the DHH children and only one parent in our sample had been infected with COVID-19 at the time of the survey; however, approximately 40% of children were at least slightly worried about becoming infected.

### 3.3. Daily Behaviors

#### 3.3.1. Changes to Child Learning or Care Arrangements

Most children (88.5%) were participating in a formal care or an education program during the pandemic (e.g., childcare, kindergarten, primary or high school), commonly a mainstream program (84% in a mainstream preschool, 69% in mainstream school, 17% in mainstream school with hearing loss special unit, and 4.5% in mainstream preschool with a special program; Supplementary Table S1). The remaining students were enrolled in schools/preschools specifically for children with hearing loss or other disabilities.

**Figure 1:**
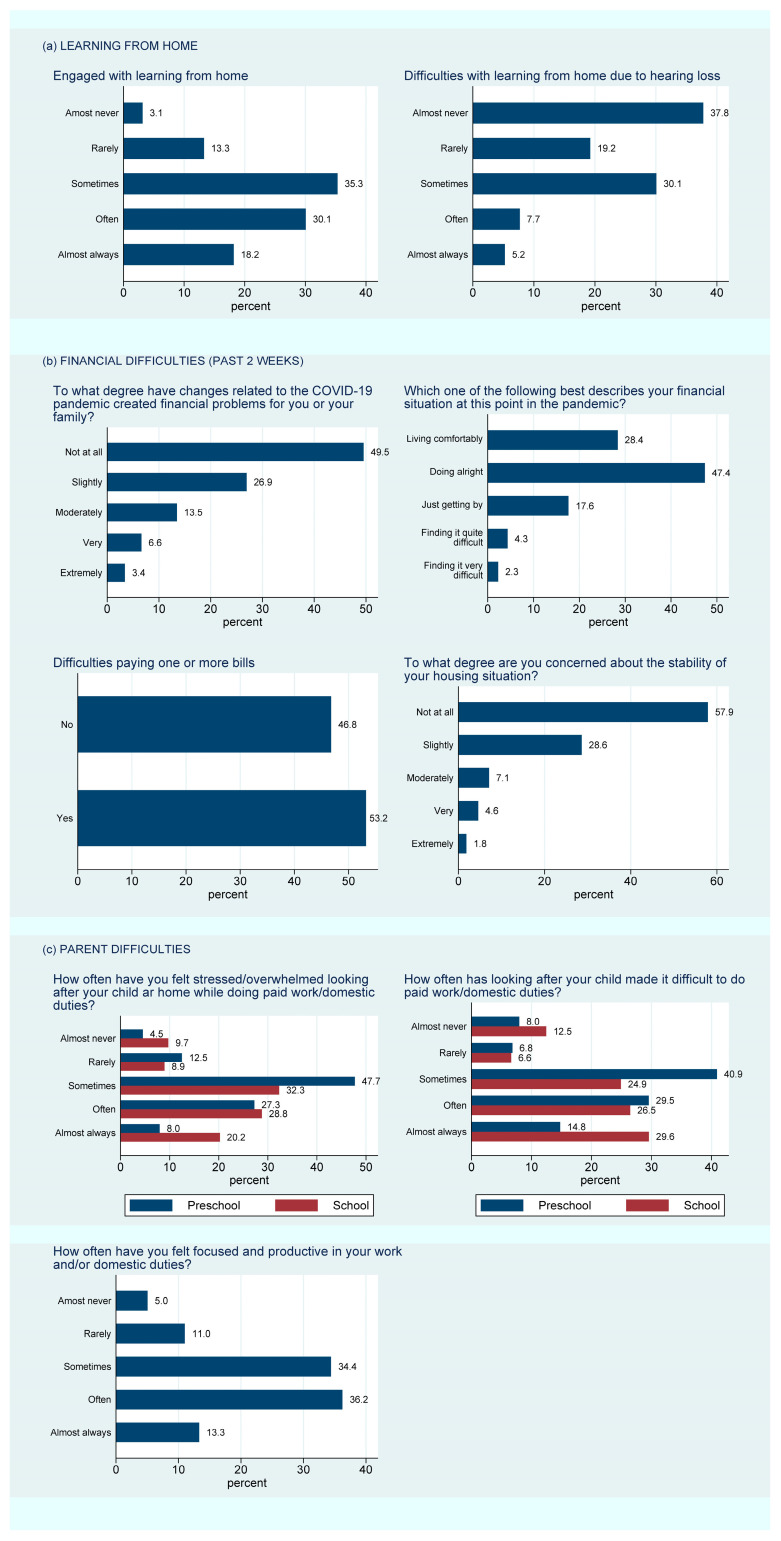
Distribution of parent responses to survey questions about (**a**) learning from home, (**b**) financial difficulties, and (**c**) parent difficulties during the COVID-19 pandemic.

Approximately 50% of parents said their child’s preschool education arrangements had changed since the pandemic started (Supplementary Table S1). Most parents of preschool children (86%) reported that the pandemic made it difficult to do paid work or domestic duties at least some of the time (Figure 1c) and 83% reported feeling stressed or overwhelmed looking after their child whilst doing their paid work or domestic duties during the pandemic (Figure 1c). In parents of primary and secondary school students, the results were similar, although a larger proportion had experienced changes to their child’s learning arrangements (~80%). Of these, 84% of parents reported that classes were being conducted remotely, with 94% of children completing assignments at home. Almost all children in our sample (99%) had easy access to the internet and a computer device, and 77% had contact with their teacher and classmates by phone or video. Nonetheless, most parents (81%) reported that tending to their child’s learning at home made it difficult to complete paid work or domestic duties at least some of the time. Parents felt stressed/overwhelmed at least some of the time (81%) and 16% rarely or almost never felt focused and productive in their work and/or domestic duties.

Despite the difficulties for parents of supervising their child’s education along with completing their normal activities, most parents (83%) reported that their child enjoyed and engaged with their learning-from-home activities at least sometimes (Figure 1a). However, almost half (43%) felt that it had been difficult, at least sometimes, for their child to participate in learning from home because of their hearing loss.

#### 3.3.2. Changes to Parent Employment

Under half of respondents (43.5%) reported that they were working for pay, with just over half of those who responded working from home (54.7%) at the time of completing the survey. Only 23.7% of respondents were working their usual hours. Likewise, less than half of partners were working for pay (46.9%), but about two-thirds (61.2%) of those who were were still attending their workplace.

#### 3.3.3. Financial Problems

Almost a quarter (23.5%) of families reported that the COVID-19 pandemic had created at least moderate financial problems for their family (Figure 1b, Supplementary Table S1). Furthermore, 24.2% reported that their family was just getting by or finding it quite/very difficult financially, and 13.5% reported moderate or higher concern about the stability of their housing situation (Figure 1b, Supplementary Table S1).

#### 3.3.4. Daily Activities

**Figure 2:**
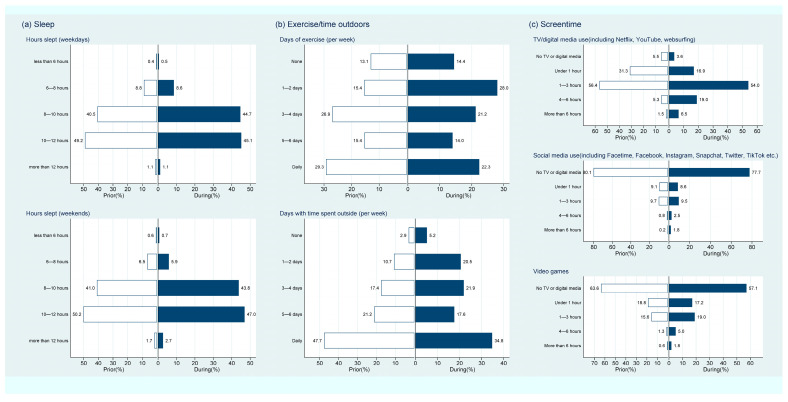
Child daily activities ((**a**) sleep, (**b**) exercise, and (**c**) screen time) prior to and during the COVID-19 pandemic.

Most children (over 90%) had more than 8 h sleep per night, both on weekends and during the week, prior to the pandemic (Figure 2a and Supplementary Table S2). This did not change substantially during the pandemic; however, bedtimes shifted later, both during the week and on the weekend, i.e., 15% had a weekend bedtime after 10 pm pre-pandemic versus 20% during the pandemic. Compared to before the pandemic, children were less active, with a shift to less days of exercise (28% pre-pandemic vs. 42.5% during the pandemic exercising two or less days per week) and less days with time spent outside (14% vs. 25.5% spending time outside two or less days, Figure 2b and Supplementary Table S2). Screen time also increased substantially with the proportion of DHH children spending less than an hour on TV/digital media decreasing from 37% to 20.5%; social media decreasing from 89% to 86%; and video games decreasing from 83% to 74% during the pandemic compared to before (Figure 2c and Supplementary Table S2).

### 3.4. Changes to Family Life

DHH children experienced significant changes to their normal life during the early stages of the COVID-19 pandemic. Almost 40% left their home for shopping, exercise, appointments, or school less than three days per week and over a third (36.5%) of children found the restrictions stressful. Despite this, most parents reported the quality of the relationship with their child was about the same (62.5%) or better (25%) than before the pandemic. However, relationships with extended family or friends were reported to be about the same (60.3%) or worse (35%), which is likely a reflection of being unable to see them in person. Most DHH children (49.4%) were very or extremely hopeful that the pandemic would end soon (Supplementary Table S1).

Like their children, 34% of parents left their homes less than three days a week and almost half (43%) found these restrictions stressful. Despite the challenges, most parents (60.6%) felt the pandemic had led to positive changes in their family and community and reported more quality time with family (47.7%) as the leading reason for their response.

### 3.5. Socio-Emotional Wellbeing of DHH Children and Their Parents

**Figure 3:**
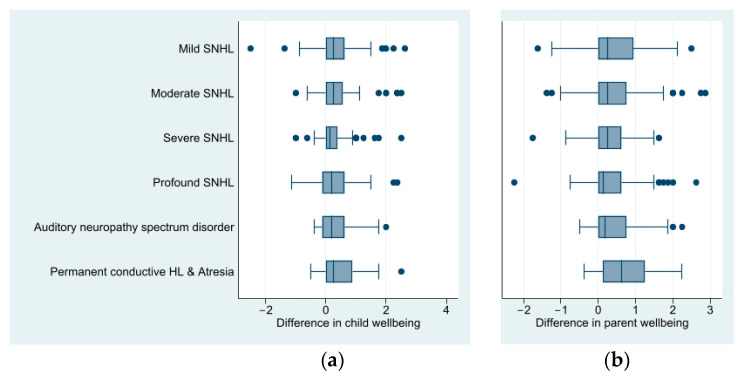
Difference in (**a**) child and (**b**) parent socio-emotional wellbeing scores during compared to before the pandemic, measured using the eight-item CRISIS emotions/worries scale, with a shift to the right indicating poorer wellbeing, i.e., a higher level of emotions/worries during the pandemic. HL: hearing loss; SNHL: sensorineural hearing loss.

Figure 3a describes the mean difference in child socio-emotional wellbeing (CRISIS emotions/worries scale eight-item score) during the pandemic compared to before. Supplementary Table S3 provides further detail, breaking hearing loss groups down further to bilateral and unilateral hearing loss. For all degrees/types of hearing loss, DHH children had poorer socio-emotional wellbeing during the pandemic compared to before the pandemic (mean emotions/worries score, EWS, change from 0.76 pre-pandemic to 1.10 during the pandemic, mean difference 0.34, 95% CI: 0.28 to 0.39). Responses were on a 5-point Likert scale between 0 and 4, with 0 representing the best outcome, i.e., least sad, and 4 representing the poorest outcome, i.e., most sad. Figure 3b summarizes the mean difference in parent CRISIS emotions/worries eight-item summary score during the pandemic compared to before, by degree/type of child hearing loss. Like their children, parent socio-emotional wellbeing was poorer during the pandemic, regardless of hearing loss degree/type (mean EWS change from 1.05 pre-pandemic to 1.43 during the pandemic, mean difference 0.38, 95% CI: 0.31 to 0.44).

In addition, 98% of parents reported that their child’s physical health was good, very good, or excellent at the time of the survey (Supplementary Table S1). A total of 97% of parents reported that their child’s mental/emotional health was good, very good, or excellent prior to the pandemic; however, parents reported that 12.5% of children were worried about the COVID-19 pandemic influencing their mental or physical health (Supplementary Table S1).

## 4. Discussion

### 4.1. Principal Findings

Parents reported that both their own and their DHH child’s socio-emotional wellbeing worsened during the pandemic compared to before, regardless of hearing loss type or severity. This occurred in the context of some DHH families experiencing significant adversity during the pandemic with 10% of parents reporting that significant financial problems were created by the pandemic, 13% were concerned about the stability of their housing situation, 24% reporting their financial situation as just getting by or finding it quite/very difficult, and 88% reporting difficulty paying at least one bill during the pandemic.

Government-mandated learning from home required parents to supervise their child’s learning while completing their own usual tasks, creating a large supervision burden, particularly for younger DHH children. In addition, just under half of parents (43%) reported that their DHH child had trouble with learning from home due to their hearing loss at least some of the time. Nonetheless, the majority (83%) reported that their DHH child was engaged with and enjoying learning from home at least some of the time. Parent experiences of learning from home were less favorable. Most parents (81%) reported feeling stressed or overwhelmed and finding it difficult at least some of the time tending to their child’s home learning while doing paid work or domestic duties. This suggests that the remote learning policy caused significant disruption and burden for the primary caregiver during this period.

### 4.2. Strengths and Limitations

This is the first and largest Australian study of its kind to document the socio-emotional experiences and wellbeing of DHH children (aged 0 to 17 years) and their parents before and during the COVID-19 pandemic. Recruitment of our study participants was from a population-based approach and data were prospectively collected. The online survey collected information about child online learning, which other studies have suggested may present a particular challenge for DHH children [21], as well as detailed data about the economic and socio-emotional consequences of the COVID-19 pandemic in DHH children throughout early life to the end of high school. Data on parent and child socio-emotional wellbeing during, compared to before, the pandemic were collected using the CRISIS survey tool, developed and validated to examine mental health/wellbeing and behavior during the COVID-19 pandemic [25]. The CRISIS tool was also used to measure socio-emotional wellbeing in other Australian child research cohorts around the same time during the COVID-19 pandemic, enabling comparison of the results from our sample with other Australian children [26,30], personal communication with Dr O’Connor dated 31 May 2023.

Limitations of this study include the parent-reported nature of all data regardless of child age. We opted to utilize a parent report format in line with our previous data collection protocols to minimize family disruption. The CRISIS instrument, from which most survey questions were drawn, is validated for parent report for children aged 5 to 17 years [25]. Nonetheless, parent-report surveys can lead to bias as the parents’ perception of their child’s life may differ from the child’s own feelings. Although VicCHILD participants were recruited through a population approach, VicCHILD families lived in areas of less disadvantage compared to the general Victorian population (SEIFA scores around 0.2 standard deviations higher than Victorian norms) [24]. This is a common bias for many research studies, where families from more disadvantaged areas are less likely to participate.

### 4.3. Interpretation in Light of Other Studies

The socio-emotional experiences we report in DHH children in Victoria, Australia, during the early stages of the COVID-19 pandemic are unique in the literature. To our knowledge, the wide range of socio-emotional experiences reported here for DHH children during the pandemic have not yet been described. Nonetheless, several studies provide useful information about the mental health outcomes of DHH children during the pandemic. Two cross-sectional analyses have reported symptoms of anxiety in Iranian and Chinese DHH adolescents [31,32]. The Iranian study included 56 adolescents aged 12 to 18 years old. It compared 23 deaf to 33 hard of hearing adolescents. In contrast to our results, this group reported higher anxiety with increased severity of hearing loss (60.9% of deaf adolescents had anxiety disorder symptoms compared to 21.2% of hard of hearing, *p* = 0.003) [32]. The Chinese sample of 13 to 27 year-olds compared children with hearing loss to those without hearing loss once the COVID-19 infection rate had stabilized below 100 cases per day [33]. They reported similar Depression Anxiety Stress Scales (DASS) stress and depression scores in individuals with hearing loss compared to those without hearing loss [31], suggesting that when infection rates are low and stable, stress and depression outcomes are similar between the two groups. Unlike our study, this study did not compare pre-pandemic to within-pandemic mental health, so no baseline data were available. However, this research suggests that all children had a similar average DASS stress and depression score during the pandemic, regardless of hearing status.

Additionally, a qualitative survey completed by 416 educational personnel who worked with DHH children in the United States between September and December 2020 demonstrated multiple challenges to the delivery of hybrid models of education during the COVID-19 pandemic. In-person education challenges specific to this cohort included difficulties in sound transmission with the use of face masks, barriers, and social distancing techniques, whereas learning from home included multiple technological challenges specific to DHH children. Changes to learning modalities often needed to be made and implemented in a time-sensitive manner which added an extra challenge to DHH families [22].

There is limited literature on the impacts of the COVID-19 pandemic on children with special needs [33]. A longitudinal study of Chilean schoolchildren, with data available before the pandemic (in 2018–2019) and during the early pandemic (in 2020), reported increased internalizing and externalizing problems following quarantine [34]. This study reported results for school children in their initial years of school with and without special educational needs and by high and low socioeconomic status (SES) [34]. They described an increase in socio-emotional internalizing problems, with those with special educational needs and low SES most severely affected following quarantine [34]. All groups were also affected by an increase in externalizing problems during the pandemic, however, this did not differ by special educational needs [34]. In addition, a qualitative study in Turkey aimed to determine emotional and behavioral challenges amongst children between 4 and 6 years of age [35]. This study enrolled families of 117 children with diagnoses of global developmental delay, autism, hearing impairment, or language delay who received special education for at least 3 months prior to the pandemic. Like us, they reported that most (91.7% of 24 DHH children included in sample) were negatively impacted by the pandemic, with poorer sleep (75%) and behavioral issues (40%) observed along with a significant increase in screen time. A second qualitative study of 37 Saudi Arabian parents of DHH children suggested that DHH children found distance education challenging and required additional support in this setting [20]. In our VicCHILD study, parents also reported child difficulties with remote learning. Future studies could further explore useful supports for families. In contrast to the results from 56 Iranian adolescents [32], we did not observe a link with the degree of hearing loss and mental wellbeing but rather an overall decrease in socio-emotional wellbeing for all children and parents, regardless of child hearing status [32].

## 5. Conclusions

In the first and largest study of its kind, we described the socio-emotional experiences and wellbeing of Australian DHH children and their parents before and during the COVID-19 pandemic. This paper provides insights into the economic and socio-emotional challenges faced by DHH children and their families during the adversities of the global pandemic. Our results reflect the substantial impacts to DHH children and their parents in many facets of their life, including school, work, financial, family dynamics, friendships, and socio-emotional wellbeing, in Victoria, Australia. We report significant challenges for parents in meeting the increased demands of online education; however, in the early stages of the pandemic, most children were still engaged in their learning at least some of the time. Nonetheless, parent and child socio-emotional wellbeing was on a downward trajectory in the first year of the pandemic when this survey was completed.

Our findings can be used in directing policy and services during future pandemics and lend support to avoiding lockdowns for children, especially those who are DHH. Further research, tracking parent and child outcomes as the pandemic has progressed, is needed to determine how best to detect and provide appropriate support to DHH children and their families. In addition, a better understanding of protective factors that support families to thrive in the ever-changing pandemic situation is critical to ensure the best outcomes for DHH children and their families.

## Figures and Tables

**Table 1:** Child and parent demographics and hearing loss information for responders and non-responders.

Measure	Responders n = 497 (62%)	Non-Responders n = 309 (38%)
**Demographics: parent**		
Sex: female—n (%)	413 (93%)	-
Relationship to child: mother—n (%)	414 (94%)	-
Partner living with parent—n (%)	399 (90%)	-
Located within Australia—n (%)	489 (98%)	-
Maternal education—n (%) ^a^		
High school not completed	44 (11%)	46 (19%)
High school completed	126 (31%)	86 (36%)
Tertiary qualification completed	240 (59%)	109 (45%)
**Demographics: child**		
Age, years—mean (SD)	6.5 (4.0)	6.9 (3.7)
Sex: female—n (%)	215 (43%)	148 (48%)
**Demographics: family**		
SEIFA disadvantage index—mean (SD) ^b^	1013.5 (65.1)	1006 (68.5)
Remoteness area: regional or remote—n (%) ^c^	96 (19%)	55 (18%)
Language spoken at home: English—n (%)	363 (73%)	214 (75%)
**Hearing loss information of child**		
Hearing loss laterality—n (%)		
Unilateral	143 (29%)	84 (27%)
Bilateral	320 (64%)	204 (66%)
Unknown	34 (7%)	21 (7%)
Hearing loss type—n (%) ^d^		
Sensorineural	339 (68%)	215 (70%)
Mixed	49 (10%)	30 (10%)
Auditory neuropathy sensory disorder	39 (8%)	27 (9%)
Atresia	19 (4%)	4 (1%)
Permanent conductive	14 (3%)	11 (4%)
Unknown	37 (7%)	22 (7%)
Degree of hearing loss—n (%)		
Mild	117 (24%)	74 (24%)
Moderate	145 (29%)	83 (27%)
Severe	84 (17%)	55 (18%)
Profound	109 (22%)	76 (25%)
Missing/ANSD (not recorded)	42 (8%)	21 (7%)

ANSD: auditory neuropathy spectrum disorder; n: sample size; SD: standard deviation; SEIFA: Socio-Economic Indexes for Areas—Index of Relative Socio-Economic Disadvantage. ^a^: Maternal education data utilized from earlier data collection so data are incomplete (data missing from 87 responders and 68 non-responders); ^b^: SEIFA is a composite census-based measure summarizing the social and economic conditions of Australian neighborhoods (national mean 1000, SD 100, where higher values represent less disadvantage); [27] ^c^: Remoteness Area summarized using Australian Standard Geographical Classification (ASGR) Remoteness Area Correspondences [28]; ^d^: in better hearing ear if hearing loss is bilateral and worse hearing ear if hearing loss is unilateral.

## Data Availability

All available data supporting reported results can be found within this publication and its Supplementary Materials. Data are not publicly available because not all VicCHILD participants have provided consent for data sharing, and data sharing is limited to ethically approved research.

## Supplementary Materials

The following supporting information can be downloaded at https://www.mdpi.com/article/10.3390/children10071147/s1, Table S1: Child and family health, education, social, and economic changes during the COVID-19 pandemic; Table S2: Daily activities prior to and during the COVID-19 pandemic; Table S3: Child and parent socio-emotional wellbeing scores by degree and laterality of child hearing loss.
