# Supplementary material for: Socio-Emotional Experiences and Wellbeing of Deaf and Hard of Hearing Children and Their Parents before and during the COVID-19 Pandemic
Source: Children (Basel). 2023 Jun 30;10(7):1147. doi: 10.3390/children10071147 (PMC10378092; doi:10.3390/children10071147)
Supplement: Supplementary file 1 [file children-10-01147-s001.zip › children-2453167-supplementary.pdf]

Supplementary Table S1: Child and family health, education, social and economic changes during the COVID-19 pandemic

Measure	Number of complete responses	Responses n=497
Child		
**Health**		
Global physical health - n (%)	490	
Excellent		221 (45.1)
Very good		214 (43.7)
Good		47 (9.6)
Fair		5 (1.0)
Poor		3 (0.6)
Global mental/emotional health prior to pandemic - n (%)	490	
Excellent		189 (38.6)
Very good		221 (45.1)
Good		66 (13.5)
Fair		12 (2.5)
Poor		2 (0.4)
COVID-19 infection – n (%)	490	0 (0)
**Education**	471	
Not in formal care/education		54 (11.5)
Childcare, family day care, pre-school		102 (22.0)
4-year old kindergarten program		46 (10.0)
Primary school		222 (47.0)
High school		46 (10.0)
Vocational		1 (0.5)
School type	269	
Mainstream		186 (69.0)
Mainstream school with HL special unit		46 (17.0)
School for children with HL		10 (4.0)
Special school for children with disabilities		23 (8.5)
Other		4 (1.5)
Preschool type	148	
Mainstream program		124 (84.0)
Mainstream with special program		7 (4.5)
Setting for children with HL		13 (9.0)
Other		4 (2.5)
**Education changes/impacts (pre-schooler's)**		
Preschool learning arrangements have changed, n (%)	148	
No, continues to attend childcare/kinder		60 (40.5)
No, continues to stay at home		12 (8.1)
Yes, was at childcare/kinder, is now at home		31 (21.0)
Yes, was at home, has returned to childcare/kinder		45 (30.4)
How often has looking after your child made it difficult to do paid work/domestic duties (pre-schooler's)?	88	
Almost never		7 (8.0)
Rarely		6 (6.8)
Sometimes		36 (40.9)
Often		26 (30.0)
Almost always		13 (14.8)

How often have you felt stressed or overwhelmed looking after your child at home while doing paid work/domestic duties (pre-schooler's)?	88	
Almost never		4 (4.6)
Rarely		11 (12.5)
Sometimes		42 (47.7)
Often		24 (27.3)
Almost always		7 (8.0)
**Education changes/impacts (primary/secondary school)**	269	
Child's primary or secondary school learning arrangements have changed		
No, has continued to attend their primary or secondary school		12 (4.5)
No, continues to learn at home		41 (15.2)
Yes, is now learning at home		77 (28.6)
Yes, was learning at home but has now returned to school		139 (51.7)
Usual classes are session	151	128 (84.8)
Child doing remote learning while being supervised at school	151	26 (17.2)
Classes being conducted remotely	118	99 (83.9)
There are assignments for child to complete	118	111 (94.1)
Child has easy access to the internet and a computer/device	118	117 (99.1)
Child has contact with their teacher by phone/video	118	91 (77.1)
Child has contact with classmates by phone/video	118	91 (77.1)
**Child education impacts**		
How much did/does your child enjoy and engage with his/her learning from home activities?	286	9 (3.2)
Almost never		38 (13.3)
Rarely		101 (35.3)
Sometimes		86 (30.1)
Often		52 (18.2)
Almost always		
Has it been difficult for your child to participate in learning from home because of his/her hearing loss?	286	
Almost never		108 (37.8)
Rarely		55 (19.2)
Sometimes		86 (30.1)
Often		22 (7.7)
Almost always		15 (5.2)
**Child social changes**		
Number of non-household contacts child had in-person conversation with in last 2 weeks, mean (SD) [Range]	449	7.9 (8.2) [0-40]
How much time has your child spent going outside of the home (e.g., shopping, exercise, appointments, school etc.)?	449	
Not at all		61 (13.6)
1-2 days per week		115 (25.6)
A few days per week		72 (16.0)
Several days per week		90 (20.0)
Every day		102 (22.7)
Several times a day		9 (2.0)

How stressful have the restrictions on leaving home been for your child?	449	
Not at all		141 (31.4)
Slightly		144 (32.1)
Moderately		103 (22.9)
Very		41 (9.1)
Extremely		20 (4.5)
Have your child's contacts with people outside of your home changed relative to before the Coronavirus/COVID-19 pandemic?	449	
A lot less		182 (40.5)
A little less		64 (14.3)
About the same		53 (11.8)
A little more		57 (12.7)
A lot more		93 (20.7)
How much difficulty has your child had following the recommendations for keeping away from close contact with people?	449	
None		103 (22.9)
A little		143 (31.9)
Moderate		99 (22.1)
A lot		63 (14.0)
A great amount		41 (9.1)
Has the quality of the relationship between you and your child changed?	448	
A lot worse		5 (1.1)
A little worse		51 (11.4)
About the same		280 (62.5)
A little better		77 (17.2)
A lot better		35 (7.8)
How stressful have these changes in family relationships been for your child?	448	
Not at all		221 (49.3)
Slightly		128 (28.6)
Moderately		55 (12.3)
Very		28 (6.3)
Extremely		16 (3.6)
Has the quality of your child's relationships with his/her friends or extended family changed?	448	
A lot worse		27 (6.0)
A little worse		133 (29.7)
About the same		270 (60.3)
A little better		10 (2.2)
A lot better		8 (1.8)
How stressful have these changes in social relationships been for your child?	448	
Not at all		162 (36.2)
Slightly		161 (35.9)
Moderately		76 (17.0)
Very		38 (8.5)
Extremely		11 (2.5)

How much has cancellation of important events (such as birthdays, holidays etc.) in your child's life been difficult for him/her?	448	
Not at all		113 (25.2)
Slightly		151 (33.7)
Moderately		102 (22.8)
Very		60 (13.4)
Extremely		22 (4.9)
To what degree is your child concerned about the stability of your living situation?	257	
Not at all		188 (73.2)
Slightly		39 (15.2)
Moderately		21 (8.2)
Very		6 (2.3)
Extremely		3 (1.2)
Did your child worry whether your food would run out because of a lack of money?	257	
Yes		17 (6.6)
No		240 (93.4)
How hopeful is your child that the COVID-19 pandemic, and the resulting changes to daily life will end soon?	257	
Not at all		25 (9.7)
Slightly		34 (13.2)
Moderately		71 (27.6)
Very		89 (34.6)
Extremely		38 (14.8)
**Child worries/changes in last 2 weeks (primary/high school)**		
Child worried about becoming infected	296	
Not at all		177 (59.8)
Slightly		87 (29.4)
Moderately		24 (8.1)
Very		8 (2.7)
Extremely		0 (0)
Child worried about friends or family being infected	296	
Not at all		173 (58.5)
Slightly		86 (29.1)
Moderately		25 (8.5)
Very		11 (3.7)
Extremely		1 (0.3)
Child worried about their physical health being influenced by Coronavirus/COVID-19?	296	
Not at all		217 (73.3)
Slightly		53 (17.9)
Moderately		18 (16.1)
Very		8 (2.7)
Extremely		0 (0)
Child worried about their Mental/Emotional health being influenced by Coronavirus/COVID-19?	296	
Not at all		190 (64.2)
Slightly		69 (23.3)
Moderately		29 (9.8)
Very		5 (1.7)
Extremely		3 (1.0)

How much is your child asking questions, reading, or talking about Coronavirus/COVID-19?	296	
Never		42 (14.2)
Rarely		79 (26.7)
Occasionally		120 (40.5)
Often		48 (16.2)
Most of the time		7 (2.4)
Has the COVID-19 pandemic, and the resulting changes to daily life led to any positive changes in your child's life?	443	
None		125 (28.2)
Only a few		176 (39.7)
Some		142 (32.1)
Parent/family		
Affected by bushfires in 2019/2020 summer		
Yes		6 (1.0)
No, but extended family were		22 (5.0)
No		414 (94.0)
Parent COVID-19 infection suspected	490	
No		442 (90.2%)
Yes, waiting for test result		1 (0.2%)
Yes, a positive test result		1 (0.2%)
Yes, a negative test result		44 (9%)
Rather not say		2 (0.4%)
Global physical health - mean (SD)	316	
Excellent		89 (28.2)
Very good		134 (42.2)
Good		80 (25.3)
Fair		12 (3.8)
Poor		1 (0.3)
Number of people currently living in home - mean (SD)	442	4.3 (1.1)
**Parent difficulties**		
Tending to child's learning at home made it difficult to do paid work and/or domestic duties	257	
Almost never		32 (12.5)
Rarely		17 (6.6)
Sometimes		64 (24.9)
Often		68 (26.5)
Almost always		76 (29.6)
How often did you feel stressed or overwhelmed tending to your child's learning at home while doing paid work/domestic duties?	257	
Almost never		25 (9.7)
Rarely		23 (9.0)
Sometimes		83 (32.3)
Often		74 (28.8)
Almost always		52 (20.2)
**Social changes**		
Number of non-household contacts had in-person conversation with in last 2 weeks - mean (SD) [Range]	438	11.2 (16.3) [0-200]

Time spent going outside of the home (e.g., shopping, exercise, appointments, work, school etc.)	440	
Not at all		17 (3.9)
1-2 days per week		137 (31.1)
A few days per week		90 (20.5)
Several days per week		82 (18.6)
Every day		107 (24.3)
Several times a day		7 (1.6)
How stressful have the restrictions on leaving home been?	440	
Not at all		71 (16.1)
Slightly		180 (40.9)
Moderately		107 (24.3)
Very		62 (14.1)
Extremely		20 (4.6)
Have your contacts with people outside of your home changed relative to before the Coronavirus/COVID-19 pandemic?	439	
A lot less		167 (38.0)
A little less		83 (18.9)
About the same		66 (15.0)
A little more		50 (11.4)
A lot more		73 (16.6)
How much difficulty have you had following the recommendations for keeping away from close contact with people?	439	
None		156 (35.5)
A little		167 (38.0)
Moderate		64 (14.6)
A lot		44 (10.0)
A great amount		8 (1.8)
Has the quality of the relationship between you and your partner changed?	439	
A lot worse		14 (3.2)
A little worse		60 (13.7)
About the same		285 (64.9)
A little better		59 (13.4)
A lot better		21 (4.8)
How stressful have these changes in your relationship been for you?	439	
Not at all		230 (52.4)
Slightly		114 (26.0)
Moderately		64 (14.6)
Very		19 (14.3)
Extremely		12 (2.7)
Has the quality of the relationship between you and your children changed?	439	
A lot worse		7 (1.6)
A little worse		50 (11.4)
About the same		238 (54.2)
A little better		114 (26.0)
A lot better		30 (6.8)

How stressful have these changes in family relationships been for you?	439	
Not at all		211 (48.1)
Slightly		128 (29.2)
Moderately		67 (15.3)
Very		23 (5.2)
Extremely		10 (2.3)
Has the quality of your relationships with friends or extended family changed?	439	
A lot worse		12 (2.7)
A little worse		119 (27.1)
About the same		283 (64.5)
A little better		19 (4.3)
A lot better		6 (1.4)
How stressful have these changes in social contacts been for you?	439	
Not at all		153 (34.9)
Slightly		150 (34.2)
Moderately		93 (23.2)
Very		36 (8.2)
Extremely		7 (1.6)
**Positive social changes**		
How much has the COVID-19 pandemic, and the resulting changes to daily life, led to positive changes in your family and your community?	439	
Not at all		50 (11.4)
Very little		123 (28.0)
Somewhat		192 (43.7)
Quite a lot		63 (14.4)
Very much		11 (2.5)
Why?	497	
More quality time with family		237 (47.7)
Less busy		152 (30.6)
People looking out for each other		110 (22.1)
Greater community outreach		52 (10.5)
Environment less polluted		133 (26.8)
Other		26 (5.2)
How hopeful are you that the COVID-19 pandemic, and the resulting changes to daily life, will end soon?	439	
Not at all		35 (8.0)
Slightly		97 (22.1)
Moderately		150 (34.2)
Very		100 (22.8)
Extremely		57 (13.0)
**Changes to work and family finances**		

Are you currently working or studying? (select all that apply)	497	
Working for pay		216 (43.5)
On leave		27 (5.4)
Working usual hours		118 (23.7)
Working reduced hours during the COVID-19 pandemic		49 (9.9)
Stood down from work during the COVID-19 pandemic		19 (3.8)
Lost job during the COVID-19 pandemic		9 (1.8)
Unemployed and looking for a job		14 (2.8)
Retired		0 (0)
Staying at home / homemaker		121 (24.4)
Disabled		1 (0.2)
Enrolled in school/college/university		32 (6.4)
Where are you working? (Select all that apply)	247	
Still going to my workplace		112 (45.3)
Working from home		135 (54.7)
Is your partner currently working or studying? (select all that apply)	497	
Working for pay		233 (46.9)
On leave		3 (0.6)
Working usual hours		160 (32.2)
Working reduced hours during the COVID-19 pandemic		58 (11.7)
Stood down from work during the COVID-19 pandemic		16 (3.2)
Lost job during the COVID-19 pandemic		11 (2.2)
Unemployed and looking for a job		11 (2.2)
Retired		1 (0.2)
Staying at home / homemaker		10 (2.0)
Disabled		3 (0.6)
Enrolled in school/college/university		4 (0.8)
Where is your partner working? (Select all that apply)	260	
Still going to their workplace		159 (61.2)
Working from home		101 (38.8)
**Financial difficulties (past 2 weeks)**		
To what degree have changes related to the Coronavirus/COVID-19 pandemic created financial problems for you or your family?	438	
Not at all		217 (49.5)
Slightly		118 (26.9)
Moderately		59 (13.5)
Very		29 (6.6)
Extremely		15 (3.4)
Which one of the following best describes your financial situation at this point in the pandemic?	437	
Living comfortably		124 (28.4)
Doing alright		207 (47.4)
Just getting by		77 (17.6)
Finding it quite difficult		19 (4.3)
Finding it very difficult		10 (2.3)
Have you had difficulties paying: (select all that apply)?	497	
Mortgage or rent or other loan re-payments		61 (12.3)
Household bills (utilities/phone/internet)		67 (13.5)
Food		29 (5.8)
Healthcare/Prescription medications		17 (3.4)
Home, car or other insurance		41 (8.3)
No difficulties		338 (68.0)
Difficulties paying one or more bills	497	438 (88.1)

Number of bills with difficulty paying	497	
0		59 (11.9)
1		374 (75.3)
2		33 (6.6)
3		17 (3.4)
4		8 (1.6)
5		6 (1.2)
To what degree are you concerned about the stability of your housing situation?	437	
Not at all		253 (57.9)
Slightly		125 (28.6)
Moderately		31 (7.1)
Very		20 (4.6)
Extremely		8 (1.8)
How often have you felt focused and productive in your work and/or domestic duties?	436	
Almost never		22 (5.1)
Rarely		48 (11.0)
Sometimes		150 (34.4)
Often		158 (36.2)
Almost always		58 (13.3)

All values are presented as the number (n) and percent of respondents (%) who selected each response for categorical variables.

Supplementary Table S2: Daily activities prior to and during the COVID-19 pandemic

Measure	n	Prior to pandemic	During pandemic	P*
**Sleep**				
Hours slept (weekdays)<br>Less than 6 hours<br>6-8 hours<br>8-10 hours<br>10-12 hours<br>12+ hours	476	2 (0.5%)<br>42 (9.0%)<br>193 (40.5%)<br>234 (49%)<br>5 (1%)	2 (0.0%)<br>38 (8.5%)<br>198 (44.5%)<br>200 (45.0%)<br>12 (2.5%)	0.23
Hours slept (weekends)<br>Less than 6 hours<br>6-8 hours<br>8-10 hours<br>10-12 hours<br>12+ hours	476	3 (0.5%)<br>31 (6.5%)<br>195 (41%)<br>239 (50%)<br>8 (2%)	3 (0.5%)<br>26 (6.0%)<br>194 (44.0%)<br>208 (47.0%)<br>12 (2.5%)	0.12
Bedtime (weekdays)<br>Before 8pm<br>8pm-10pm<br>10pm-12am<br>After midnight	476	203 (43%)<br>247 (52%)<br>25 (5%)<br>1 (0%)	142 (32%)<br>258 (58%)<br>37 (8%)<br>6 (2%)	<0.0001
Bedtime (weekends)<br>Before 8pm<br>8pm-10pm<br>10pm-12am<br>After midnight	476	136 (28%)<br>271 (57%)<br>60 (13%)<br>9 (2%)	114 (26%)<br>241 (54%)<br>76 (17%)<br>12 (3%)	<0.0001
**Exercise**				
Days of exercise (per week)<br>None<br>1-2 days<br>3-4 days<br>5-6 days<br>Daily	475	62 (13%)<br>73 (15%)<br>128 (27%)<br>73 (15%)<br>139 (29%)	64 (14.5%)<br>124 (28%)<br>94 (21%)<br>62 (14%)<br>99 (22.5%)	<0.0001
Days with time spent outside (per week)<br>None<br>1-2 days<br>3-4 days<br>5-6 days<br>Daily	476	14 (3%)<br>51 (11%)<br>83 (17%)<br>101 (21%)<br>227 (48%)	23 (5%)<br>91 (20.5%)<br>97 (22%)<br>78 (17.5%)<br>154 (35%)	<0.0001
**Screen time**				
**TV/digital media use** (including Netflix, YouTube, web surfing)<br>None<br>Under 1 hour<br>1-3 hours<br>4-6 hours<br>6 or more hours	473	26 (5.5%)<br>148 (31.5%)<br>267 (56.5%)<br>25 (5.5%)<br>7 (1.5%)	16 (3.5%)<br>75 (17%)<br>239 (54%)<br>84 (19%)<br>29 (6.5%)	<0.0001

**Social media use** (including Facetime, Facebook, Instagram, Snapchat, Twitter, TikTok etc.)	473			0.0001
None		379 (80%)	344 (77.5%)	
Under 1 hour		43 (9%)	38 (8.5%)	
1-3 hours		46 (9.5%)	42 (9.5%)	
4-6 hours		4 (1%)	11 (2.5%)	
6 or more hours		1 (0.5%)	8 (2%)	
**Video games**	473			<0.0001
None		301 (64%)	253 (57%)	
Under 1 hour		89 (19%)	76 (17%)	
1-3 hours		74 (15.5%)	84 (19%)	
4-6 hours		6 (1%)	22 (5%)	
6 or more hours		3 (0.5%)	8 (2%)	

All values are presented as the number (n) and percent of respondents (%) who selected each response for categorical variables. A Stuart Maxwell test was used to test whether distributions are the same for categorical variables.

Supplementary Table S3: Mean child and parent socio-emotional wellbeing scores by degree and laterality of child hearing loss

Mean wellbeing score*	Pre-pandemic			During pandemic		Difference between pre and during pandemic		
	n	Mean	95% CI	Mean	95% CI	Mean difference	95% CI	p
**Child**								
Mild	101	0.83	0.70 to 0.96	1.21	1.06 to 1.37	0.38	0.25 to 0.52	<0.01
Moderate	118	0.72	0.61 to 0.82	1.06	0.92 to 1.21	0.35	0.24 to 0.45	<0.01
Severe	69	0.80	0.65 to 0.94	1.04	0.88 to 1.20	0.24	0.12 to 0.37	<0.01
Profound	89	0.74	0.63 to 0.84	1.05	0.88 to 1.21	0.31	0.18 to 0.44	<0.01
ANSD	34	0.58	0.47 to 0.69	0.91	0.67 to 1.16	0.33	0.13 to 0.53	<0.01
Permanent Conductive in. Atresia	29	0.91	0.69 to 1.13	1.37	1.07 to 1.66	0.46	0.18 to 0.73	<0.01
**Parent**								
Mild	100	1.10	0.98 to 1.22	1.52	1.35 to 1.68	0.42	0.28 to 0.56	<0.01
Moderate	116	1.07	0.96 to 1.18	1.41	1.26 to 1.55	0.34	0.21 to 0.47	<0.01
Severe	69	1.00	0.85 to 1.16	1.29	1.11 to 1.47	0.29	0.13 to 0.44	<0.01
Profound	88	1.02	0.88 to 1.16	1.35	1.19 to 1.51	0.33	0.19 to 0.48	<0.01
ANSD	34	1.12	0.87 to 1.37	1.52	1.19 to 1.85	0.40	0.15 to 0.65	<0.01
Permanent Conductive in. Atresia	29	1.07	0.83 to 1.31	1.75	1.39 to 2.11	0.68	0.42 to 0.94	<0.01
Bilateral hearing loss								
**Child**								
Mild	78	0.80	0.60 to 0.67	1.24	1.07 to 1.41	0.44	0.28 to 0.60	<0.01
Moderate	88	0.72	0.60 to 0.83	1.07	0.89 to 1.25	0.35	0.22 to 0.48	<0.01
Severe	47	0.78	0.59 to 0.97	1.08	0.86 to 1.29	0.30	0.14 to 0.45	<0.01
Profound	50	0.80	0.65 to 0.94	1.10	0.83 to 1.28	0.26	0.09 to 0.43	<0.01
ANSD	14	0.51	0.36 to 0.65	0.77	0.45 to 1.08	0.26	0.03 to 0.49	0.03
Permanent Conductive in. Atresia	10	0.79	0.41 to 1.16	0.95	0.42 to 1.48	0.16	-0.19 to 0.51	0.31
**Parent**								
Mild	77	1.10	0.97 to 1.24	1.59	1.42 to 1.77	0.49	0.32 to 0.66	<0.01
Moderate	86	1.04	0.90 to 1.18	1.44	1.26 to 1.62	0.40	0.24 to 0.57	<0.01
Severe	47	1.06	0.85 to 1.26	1.35	1.13 to 1.57	0.30	0.09 to 0.51	<0.01
Profound	49	1.10	0.88 to 1.31	1.42	1.21 to 1.62	0.32	0.12 to 0.52	0.02
ANSD	14	1.01	0.49 to 1.52	1.38	0.82 to 1.93	0.37	0.02 to 0.71	0.03
Permanent Conductive in. Atresia	10	1.04	0.53 to 1.54	1.46	0.83 to 2.09	0.43	-0.04 to 0.89	0.07

Unilateral hearing loss								
**Child**								
Mild	14	0.92	0.54 to 1.30	1.19	0.67 to 1.70	0.27	-0.08 to 0.62	0.12
Moderate	22	0 .74	0.46 to 1.02	1.07	0.76 to 1.39	0.34	0.12 to 0.55	<0.01
Severe	14	0.83	0.59 to 1.12	0.88	0.48 to 1.18	0.04	-0.21 to 0.30	0.71
Profound	37	0.66	0.50 to 0.81	1.04	0.77 to 1.31	0.39	0.19 to 0.58	<0.01
ANSD	19	0.63	0.45 to 0.80	0.93	0.58 to 1.29	0.31	0.01 to 0.61	0.04
Permanent Conductive in. Atresia	19	0.97	0.68 to 1.27	1.59	1.24 to 1.93	0.61	0.23 to 0.99	<0.01
**Parent**								
Mild	14	1.13	0.72 to 1.53	1.44	0.81 to 2.07	0.31	-0.04 to 0.67	0.08
Moderate	22	1.21	0.98 to 1.44	1.40	1.11 to 1.68	0.19	-0.07 to .45	0.15
Severe	14	0.86	0.55 to 1.16	1.09	0.70 to 1.48	0.23	-0.10 to 0.57	0.16
Profound	37	0.93	0.74 to 1.12	1.31	1.05 to 1.57	0.38	0.15 to 0.60	<0.01
ANSD	19	1.20	0.91 to 1.49	1.55	1.11 to 1.98	0.35	-0.02 to 0.71	0.06
Permanent Conductive in. Atresia	19	1.09	0.79 to 1.38	1.90	1.43 to 2.37	0.82	0.49 to 1.14	<0.01

*Mean wellbeing score is generated using the CRISIS emotions/worries 8-item summary score which includes 8 questions rated on a 5-point Likert scale between 0 and 4, with 0 representing the best outcome (i.e. least sad) and 4 representing the poorest outcome (i.e. most sad). P-values are from a paired t-test comparing the three months before the pandemic (pre pandemic) to the last two weeks (during the pandemic).
